# Growth of Peripheral and Central Nervous System Tumors Is Supported by Cytoplasmic c-Fos in Humans and Mice

**DOI:** 10.1371/journal.pone.0009544

**Published:** 2010-03-04

**Authors:** David C. Silvestre, Germán A. Gil, Nicolás Tomasini, Daniela F. Bussolino, Beatriz L. Caputto

**Affiliations:** Departamento de Química Biológica, Facultad de Ciencias Químicas, CIQUIBIC, Universidad Nacional de Córdoba, Córdoba, Argentina; University of Minnesota, United States of America

## Abstract

**Background:**

We have previously shown that the transcription factor c-Fos is also capable of associating to endoplasmic reticulum membranes (ER) and activating phospholipid synthesis. Herein we examined phospholipid synthesis status in brain tumors from human patients and from NPcis mice, an animal model of the human disease Neurofibromatosis Type 1 (NF1).

**Principal Findings:**

In human samples, c-Fos expression was at the limit of detection in non-pathological specimens, but was abundantly expressed associated to ER membranes in tumor cells. This was also observed in CNS of adult tumor-bearing NPcis mice but not in NPcis *fos*(−/−) KO mice. A glioblastoma multiforme and a malignant PNS tumor from a NF1 patient (MPNST) showed a 2- and 4- fold c-Fos-dependent phospholipid synthesis activation, respectively. MPNST samples also showed increased cell proliferation rates and abundant c-Fos expression.

**Conclusions:**

Results highlight a role of cytoplasmic c-Fos as an activator of phospholipid synthesis in events demanding high rates of membrane biogenesis as occurs for the exacerbated growth of tumors cells. They also disclose this protein as a potential target for controlling tumor growth in the nervous system.

## Introduction

The expression of c-Fos is tightly regulated responding rapidly and transiently to a plethora of stimuli [1]–[3]. c-Fos heterodimerizes with proteins of the *jun* family to form some of the many AP-1 transcription factors that regulate the expression of target genes such as those involved in the initiation of DNA synthesis as a response to growth factors [1], [4]. However, understanding the participation of AP-1 in complex processes such as those of multi-step tumorigenesis has not yet been completely achieved.

We have shown that, in addition to its nuclear activity, c-Fos associates to membranes of the endoplasmic reticulum (ER), the main site of phospholipid production, and activates their synthesis. This has been observed in light stimulated retina ganglion and photoreceptor cells in vivo [5], [6], in NIH 3T3 cells induced to re-enter growth [7] and in PC12 cells induced to differentiate [8], [9].

In actively growing and proliferating T98G cells, a human glioblastoma multiforme-derived cell line, it is observed abundant ER-associated c-Fos expression and c-Fos-dependent phospholipid synthesis activation, both of which are reversibly inhibited upon phosphorylation of c-Fos tyrosine residues [10]. Phosphorylation of c-Fos tyrosine residues was also observed in NIH 3T3 fibroblasts [10]. We hypothesized from these results that in cells committed to grow and/or proliferate, activation of lipid synthesis by c-Fos is crucial for enabling the endomembrane system to supply the massive demands of membrane to sustain these processes.

In the search for experimental paradigms including the complex environment in which in vivo tumor development occurs, herein we examined the status of cytoplasmic c-Fos expression and phospholipid synthesis activation in malignant human brain tumors, in the NPcis mouse -an animal model of the human disease Neurofibromatosis Type I- and also in NPcis mice KO for c-Fos. Results show that high rates of proliferation are tightly coupled to an elevated expression of ER-associated c-Fos together with activated rates of phospholipid synthesis.

## Results

### Malignant Human Brain Tumors Show Abundant Cytoplasmic c-Fos and Activated Phospholipid Synthesis

**Figure 1:**
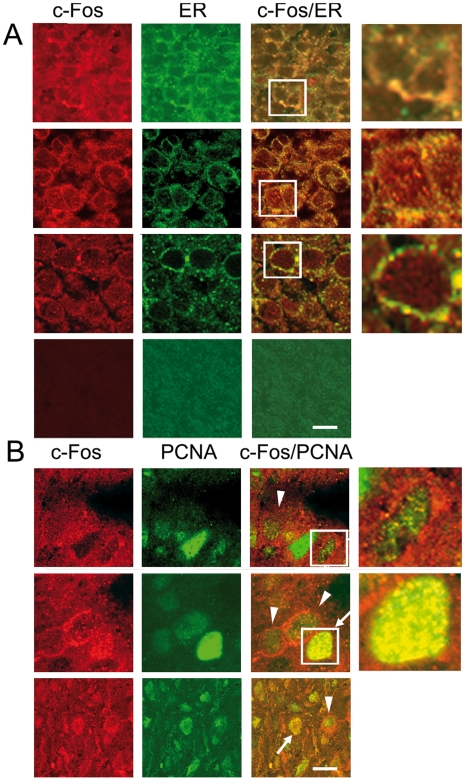
Human brain tumors show abundant c-Fos expression co-localizing with the ER marker calnexin. (**A**) Expression of c-Fos, the ER marker calnexin, and the nuclear marker of proliferating cells PCNA in human brain tumor specimens (n = 156) and non-pathological samples (n = 17). Representative samples of astrocytoma (1st row), GM (2nd row), medulloblastoma (3rd row) and human brain non-pathological samples (4th row) from a tissue array immunostained for c-Fos (red) and calnexin (green); 3rd column is the merge of the previous two columns. Yellow color evidences c-Fos/ER co-localization sites. (**B**) Immunostaining for c-Fos (red), PCNA (green) and the merged image of both is shown for an astrocytoma (1st row), a GM (2nd row) and a medulloblastoma (3rd row). Arrows: proliferating cells with nuclear c-Fos; arrowheads: cells showing predominantly peri-nuclear c-Fos. Bar: 20 µm. Fourth column in A and B is a 20× magnification of the boxed area in the 3rd column.

**Figure 2:**
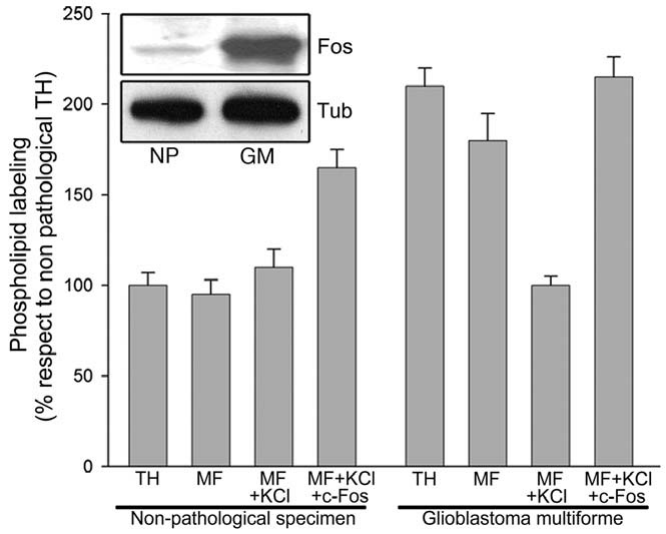
Phospholipid synthesis is activated in c-Fos-containing membranes from a human GM. A human GM and adjacent non pathological tissue excised from the same patient were used to prepare total homogenate (TH), microsomal membrane fraction (MF), 1M KCl-stripped microsomal membrane fraction (MF+KCl) and stripped MF plus 1 ng of c-Fos/µg of TH (MF+KCl+c-Fos) and assayed for phospholipid synthesis capacity. Results are the mean % ± SD of 3 experiments performed in triplicate; non-pathological TH values were taken as 100%. Inset: WB for c-Fos content in the samples assayed; Tubulin staining is used as a loading control. NP: non-pathological; GM: glioblastoma multiforme.

Since previous results in T98G cells indicate an association of cytoplasmic c-Fos expression with tumor cell proliferation and growth [10], this was examined in an array of 156 human brain tumors and 17 non-pathological tissue samples. Representative samples in Fig. 1A show abundant cytoplasmic c-Fos expression in all tumor specimens examined that included glioblastoma multiforme (GM) (n = 101), medulloblastomas (n = 15), ependymomas (n = 7) and astrocytomas (n = 33). c-Fos/ER co-localization was also found in all specimens whereas only 25% of them were positive for nuclear c-Fos (Fig. 1B). By contrast, the examination of 17 human non-pathological brain specimens showed c-Fos and the ER marker calnexin near their detection limit (Fig. 1A, 4th row).

c-Fos content and phospholipid synthesis activation were also examined in total homogenate (TH) and in microsomal fraction (MF) prepared from a non-fixed human GM and in a paired non-pathological sample (excised during surgery for tumor extirpation). Phospholipid synthesis capacity was determined *in vitro* in TH, in MF and in 1M-KCl-stripped MF prepared from these paired specimens. Phospholipid synthesis was activated only in the c-Fos-containing fractions prepared from the GM (TH and MF) but disappeared after stripping MF of associated proteins with KCl (Fig. 2). The decrease in phospholipid synthesis in KCl-stripped tumor MF was restored to initial levels by adding c-Fos to the assay medium. Phospholipid synthesis activation was also observed in the non-pathological MF upon c-Fos addition. WB examination showed abundant c-Fos expression in the GM in comparison with the non-pathological specimen which was at its detection limit (inset, Fig. 2). Similar results were obtained with 12 additional non-fixed human malignant brain tumors that included: GM (n = 9), medulloblastomas (n = 1) and astrocytomas (n = 3) (not shown).

The low immunolabeling observed for calnexin in non-pathological samples is in accordance with previous observations in which the content and concentration of ER membrane markers were found to correlate with the proliferative status of brain tissue: at early developmental stages, actively growing and dividing cells demand elevated rates of membrane biogenesis and show abundant expression of ER resident proteins and a significantly higher content of ER membranes which decreases as development proceeds. In mouse brain, c-Fos expression shows a similar pattern during development: it is high and co-localizes with the ER markers in the embryo whereas in the adult, immunostaining of both is practically undetectable ([11] and Fig. S1).

Deregulation of the ER homeostasis has been correlated with cancer in many instances [12]. Having found herein that the expression of both c-Fos and ER markers are significantly higher in tumor samples as compared to non-pathological tissue and that c-Fos is associated to the ER in all cases gives support to the need of ER-associated c-Fos for the progression of CNS tumors.

### NPcis Mice Tumors Show High Levels of c-Fos Expression

Given the results described above, the relevance of cytoplasmic c-Fos for tumor growth was validated *in vivo* in the animal model of the human syndrome Neurofibromatosis Type I (NF1), the NPcis mouse, which allows the follow-up of tumor progression with respect to c-Fos expression.

Patients suffering of NF1 are born with one mutated allele of the *nf1* gene which encodes for neurofibromin, a member of the Ras-GAP family that is particularly expressed in the CNS and PNS. As this mutated allele is non-functional, the subsequent inactivation of the remaining functional allele on a given cell type is sufficient to promote tumor formation [13], [14]. The primary clinical feature of NF1 is the development of benign peripheral nerve sheath tumors termed neurofibromas [15], which are composed primarily of neoplastic Schwann cells and non-neoplastic stromal cells. Dermal neurofibromas are benign and associated with a single nerve whereas diffuse neurofibromas involve many fascicles and are susceptible to become malignant, in which case they are denominated Malignant Peripheral Nerve Sheath Tumors (MPNST's) [16]. Between 15% and 50% of NF1 patients develop some type of glioma, although they are often indolent in nature [17].

**Figure 3:**
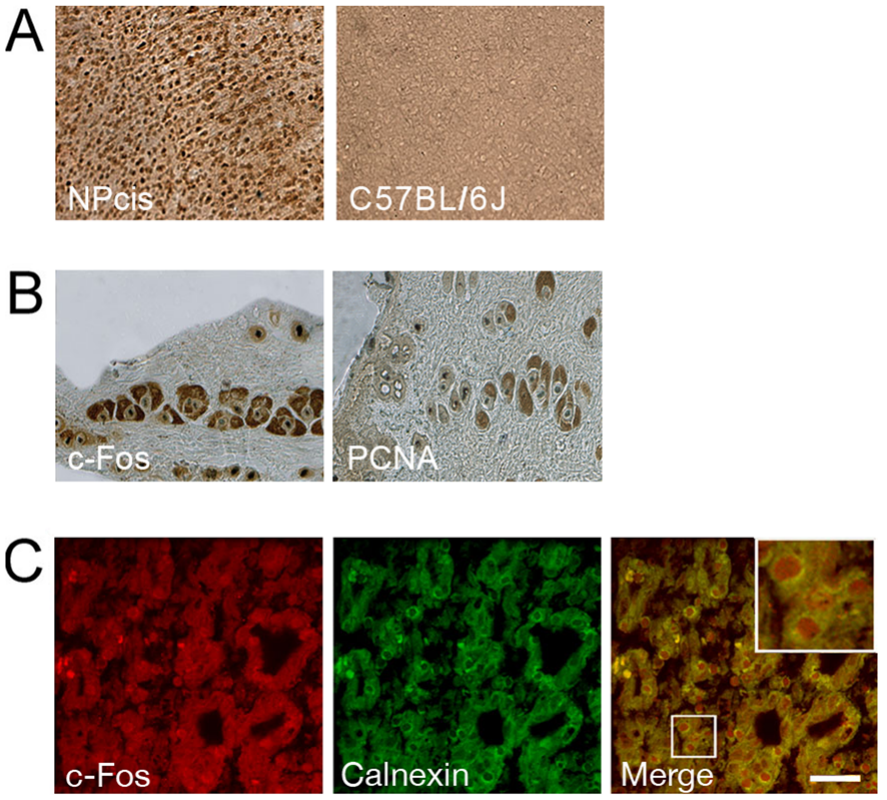
Expression and subcellular localization of c-Fos in CNS and PNS tumors from NPcis mice. (**A**) c-Fos expression was determined in mouse brain cortex from a tumor-bearing NPcis mouse (left) and a WT littermate (right) by staining with DAB-peroxidase. Note the abundant c-Fos expression in the NPcis sample, contrasting with the undetectable levels in the WT animal. (**B**) c-Fos (left) and PCNA (right) expression in peripheral nerve tumors stained as in (A). (**C**) Confocal immunofluorescence analysis of peripheral nerve tumors evidencing abundant c-Fos (red) and the ER marker Calnexin (green) immunolabeling. The merged image at right clearly shows c-Fos/ER co-localization. Three additional animals showed the same results as in (A), (B) and (C). Inset: magnification of the box delimited area. Bar: 20 µm.

NPcis mice bare, on a C57BL/6J background, a disrupted allele of both the *trp*53 and the *nf1* tumor suppressor genes that are located on chromosome 11 at 7 cM of distance from each other. Consequently, both genes usually segregate together. Loss of heterozygosis determines the spontaneous development of CNS and PNS tumors with a penetrance close to 100% by the age of 6-7 months [18]. The histological pattern of the CNS tumors resembles that of GM whereas the PNS tumors show the histological characteristics of human MPNST's [19], [20]. These animals have been proposed as a good model to study secondary glioblastomas that in humans progress from lower grades of astrocytomas involving loss of TP53 [18].

c-Fos expression was examined in CNS and PNS tumors from 6-month-old NPcis mice with clear signs of PNS tumor burden and compared to littermate C57BL/6J wild type (WT) animals. As expected, high levels of c-Fos were observed in brain cortex from NPcis animals (Fig. 3A, left panel) contrasting with the lack of expression in the same area of their WT littermates (Fig. 3A, right panel). PNS tumor samples also clearly show abundant c-Fos and the proliferation marker PCNA immunostaining with a histology resembling Schwann cells (Fig. 3B). Schwann cells are thought to be the initiation site of peripheral tumor malignancy in the course of disease development [21], [22]. As expected, c-Fos immunostaining was found colocalizing with the ER marker calnexin in these PNS tumors (Fig. 3C).

### Adult NPcis Brain Show High Levels of c-Fos Expression as Compared to WT Animals

**Figure 4:**
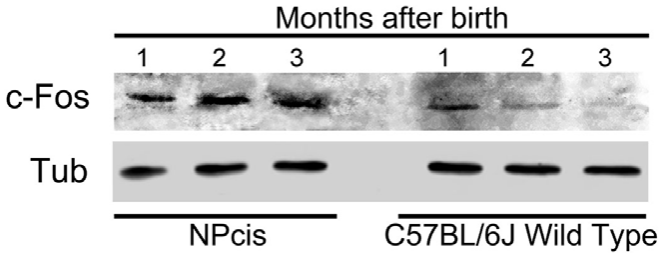
c-Fos expression in NPcis and C57BL/6J WT mice brain according to age and to the proliferative status of the tissue. c-Fos content was determined by WB in brain homogenate from NPcis and WT mice at the ages indicated (upper panel). Tubulin was used as a loading control (lower panel). Results shown are from one representative experiment out of two performed.

To correlate c-Fos expression levels with abnormal proliferation in adult NPcis brain, the content of c-Fos, determined by WB, in brain TH from NPcis mice was compared with that from their C57BL/6J WT littermates at the indicated times after birth (Fig. 4). As expected, at 1 month of age, detectable levels of c-Fos were found both in NPcis and WT brain samples which, in the latter case, decreased with age [23], [24]. By contrast, in the NPcis samples both at 2 and 3 months of age, c-Fos content remained at the levels found at 1 month or even tended to increase. These high levels of c-Fos expression observed in the NPcis animals at ages in which in WT mice proliferation has ceased and c-Fos expression declined, point to the persistence of a proliferative condition of the NPcis brain even prior to noticeable PNS tumor manifestation.

### *In Vivo* Blocking of c-Fos Expression Blocks Cell Proliferation

We next studied the dependence of proliferation in the CNS on c-Fos expression. For this, 4-month-old tumor bearing-NPcis animals were treated with a c-Fos mRNA antisense oligonucleotide (ASO), the corresponding sense (or scrambled) oligo (SO) or vehicle infused intra-cranially by means of a subcutaneously implanted osmotic pump which was connected to a cannula inserted in the caudate putamen (CP) to constantly deliver treatment solution during 28 days. The area of CP to which the pumps dispensed the treatment solution was delimited by loading pumps with 0.5% w/v methylene blue and infusing as in the experimental animals.

**Figure 5:**
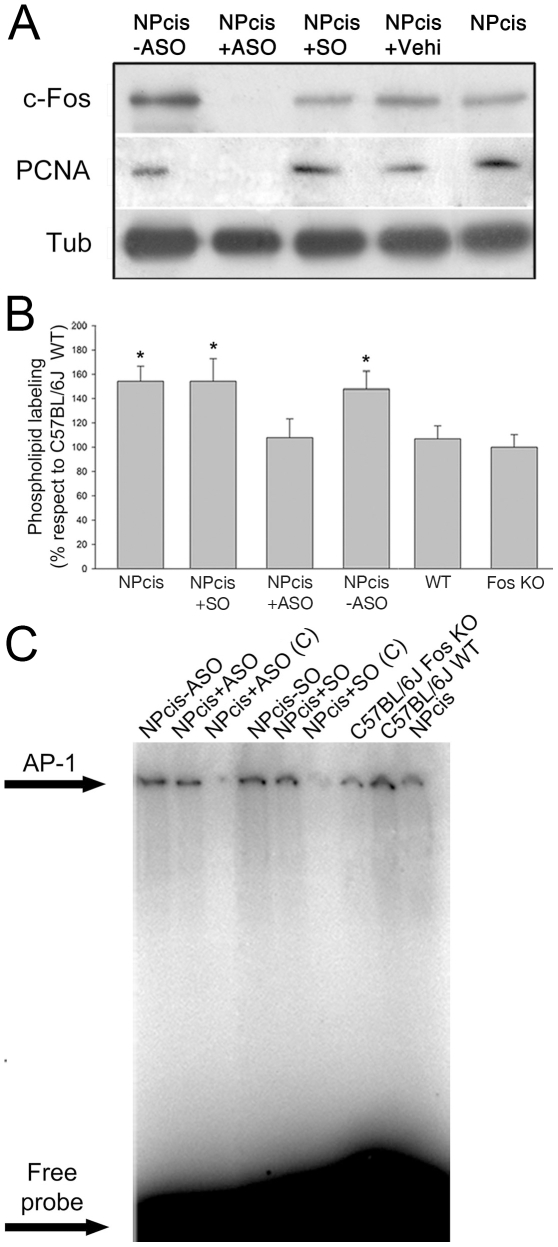
Phospholipid synthesis activation and AP-1 content in NPcis mice brain as compared to C57BL/6J WT or *fos* (−/−) KO mice. (**A**) NPcis mice at 4 months of age showing clear PNS tumor burden were treated during 28 days with ASO, SO or vehicle dispensed by an osmotic pump into the caudate putamen (CP) (see Materials and Methods). After treatment, animals were sacrificed and CP examined by WB for c-Fos and PCNA expression, the latter as an indication of the proliferative status of the tissue. The first two lanes correspond to CP from the left (non-treated) and right (ASO-treated) brain hemispheres from the same NPcis mouse. The 3rd and 4th lanes correspond to the CP from the right, SO-treated or vehicle-treated hemisphere from NPcis mice. The last lane corresponds to CP from a non-treated NPcis mouse. One experiment representative of two performed is shown. Tubulin was stained as a loading control. (**B**) Phospholipid synthesis was measured *in vitro* in CP from NPcis mice non-treated (1st column), or treated during 28 days as indicated in (A) with SO (2nd column) or from NPcis mice treated only in the right hemisphere with ASO (3rd column, NPcis+ASO) and compared to CP from the left hemisphere of the ASO-treated animals (4th column, NPcis –ASO) or to WT (5th column) or C57BL/6J *fos* (−/−) KO mice (last column), as indicated. Results are the mean ± SD of two independent experiments performed in triplicate; *p<0.005 with respect to WT mice as determined using Student's *t* test. (**C**) EMSA determination of total AP-1 content in nuclear fractions prepared from CP of mice treated as stated in (A). The first two lanes correspond to the left, non-treated and right, ASO-treated brain hemispheres from the same NPcis mouse. The 4th and 5th lanes correspond to the left, vehicle-treated and right, SO-treated hemispheres from the same NPcis mouse. The last 3 lanes correspond to C57BL/6J *fos*(−/−) KO mouse (7th lane), a C57BL/6J WT mouse (8th lane) and a non-treated NPcis mouse (last lane). Lanes marked with (C) were competed with 100X unlabeled AP-1 probe (3rd and 6th lanes) to establish binding specificity. Results are from one of three experiments performed with the same results.

Fig. 5A shows the expression levels of c-Fos and of the proliferation marker PCNA in CP determined by WB. The first 2 lanes correspond to the same NPcis animal in which the left hemisphere did not receive c-Fos ASO (NPcis-ASO) whereas the right hemisphere did (NPcis+ASO); the 3rd and 4th lanes correspond to CP from NPcis animals treated with sense oligo (NPcis+SO) or vehicle (NPcis+vehicle) respectively, whereas the 5th lane shows CP from a non-treated NPcis mouse. These results once again highlight the need of c-Fos expression for proliferation as specifically blocking it also blocks cell proliferation of tumor cells as indicated by PCNA expression (compare the 2nd lane with all other ones of Fig. 5A).

### *In Vivo* Blocking of c-Fos Expression Abrogates Phospholipid Synthesis Activation without Affecting AP1 Content

The transcriptional AP-1 activity of c-Fos has been exhaustively examined and its importance in cell proliferation well documented [1]. Consequently, it was deemed of importance to determine the relevance of both activities of c-Fos in supporting proliferation and growth of tumors: its participation in the formation of AP-1 transcription factors and its phospholipid synthesis activation capacity. To address this point, both AP-1 content and phospholipid synthesis capacity were examined *in vitro* in CP excised from 7-month-old tumor bearing-NPcis animals expressing high amounts of c-Fos (non-treated NPcis or treated with SO intra-cranially during 28 days as in Fig. 5A) or having low/null content of c-Fos (treated intra-cranially with ASO or C57BL/6J *fos* KO mice), and in untreated C57BL/6J WT animals.

Fig. 5B shows the phospholipid synthesis capacity in CP from non-treated NPcis mice (NPcis), or from NPcis mice treated as in Fig. 5A with SO (NPcis+SO) or ASO (NPcis+ASO) dispensed into the right hemisphere; NPcis-ASO corresponds to the non-treated left hemisphere of the ASO-treated animals. Clearly, blocking the expression of c-Fos with ASO abrogates phospholipid synthesis activation to similar rates as those found in WT animals (C57BL/6J WT) whereas SO has no effect. For comparative purposes, the phospholipid synthesis capacity of CP from C57BL/6J *fos* KO mice was also determined (Fig. 5B, last column) and found similar to that of WT mice (Fig. 5B, 5th column) which, as shown in Fig. 4, contain undetectable levels of c-Fos.

Next, we determined if blocking c-Fos expression affects the bulk content of AP-1 transcription factor dimers by electrophoresis mobility shift assays (EMSA) performed with nuclear extracts prepared from the brain samples used in Fig. 5B. Interestingly, no significant differences were observed in the AP-1 content of any of the different experimental conditions examined, that is, in those containing abundant c-Fos (lanes 1, 3 and 4) or those lacking appreciable amounts of c-Fos as is the case of the NPcis+ASO mice (lane 2), C57BL/6J WT or *fos* KO mice (lanes 7 and 8)(Fig. 5C). It is interesting to note that the results shown in the first two lanes of Fig. 5C (NPcis-ASO and NPcis+ASO) correspond to the left and right hemispheres of the same NPcis animals that were treated with ASO only in the right one and, as shown in Fig. 5B, differ significantly in their capacity to activate phospholipid synthesis that is tightly correlated to their c-Fos content but not in their AP-1 content as shown in Fig. 5C.

That the total levels of AP-1 result roughly constant irrespective of the presence or absence of c-Fos (including that in the *fos* KO mice) points to the presence of other, c-Fos-devoid, AP-1 dimers. Taken together, these results highlight the capacity of c-Fos to activate phospholipid synthesis as relevant to sustain proliferation and growth of adult brain tumor cells.

### NPcis *fos* (−/−) Mice Show No PNS Tumors

To further examine the importance of c-Fos expression for the generation of PNS tumors in NPcis mice, NPcis mice with germline disruptions on both c-Fos alleles were obtained (see Materials and Methods) and PNS tumor incidence examined. C57BL/6J *fos*(−/−) mice, although viable, die at approximately 7 months of age, are infertile and growth-retarded respect to their WT littermates [25], [26]. NPcis, *fos*(−/−) animals displayed these phenotypes, whereas NPcis, *fos*(+/−) were found indistinguishable from their NPcis, *fos*(+/+) counterpart mice.

Peripheral tumor incidence was determined in NPcis, *fos*(−/−) mice at the time of their death and compared to their NPcis, *fos*(+/+) or (+/−) littermates. Table 1 shows that PNS tumor formation was not observed in any of the NPcis, *fos*(−/−) mice born from independent mothers whereas the NPcis, *fos*(+/+) or *fos*(+/−) animals developed neurofibromas with an incidence similar to that observed previously [18] at the ages considered in each case (mean incidence of 71.4% animals with tumor burden at the age of 4–5 months contrasting with 0% incidence in their NPcis, *fos*(−/−) littermates). So, no PNS tumor development could be observed when c-Fos expression is intrinsically impeded by interrupting its gene in the NPcis mice whereas no differences were observed in tumor formation penetrance between the NPcis *fos*(+/+) and *fos*(−/+) mice.

**Table 1:** NPcis, *fos*(−/−) mice do not generate tumors.

	NPcis, *fos*(−/−)		Littermates		[NPcis, fos(+/+) and fos(+/−)]	
Mouse	lifespan	tumor	littermate	tumor	age (days) when	% with
(N°)	(days)	burden	(N°)	burden	tumor was detected	tumor
1	100	no	#1	no		
			#2	yes	v.a.s.*	50%
2	120	no	#1	no		
			#2	yes	v.a.s.	
			#3	yes	69	66%
3	123	no	#1	yes	99	100%
4	141	no	#1	no		
			#2	yes	v.a.s.	
			#3	yes	116	66%
5	146	no	no NPcis littermates			
6	166	no	#1	no		
			#2	yes	v.a.s.	
			#3	yes	148	
			#4	yes	155	75%

Tumor burden in all NPcis littermates was determined (or confirmed in the case detection was prior to sacrifice) at the time of death of the NPcis, *fos*(−/−) mice. No differences were observed in tumor burden between NPcis, *fos*(+/+) and NPcis, *fos*(+/−) mice which were pooled as a single group and compared to their NPcis, *fos*(−/−) littermate. Mean tumor incidence in the NPcis, *fos*(+/+) plus NPcis, *fos*(+/−) animals was 71.4% (n = 13) contrasting with the 0% (n = 6) found in the NPcis, *fos*(−/−) group.

*v.a.s.: verified at sacrifice.

### c-Fos Expression in a Human PNS Tumor

**Figure 6:**
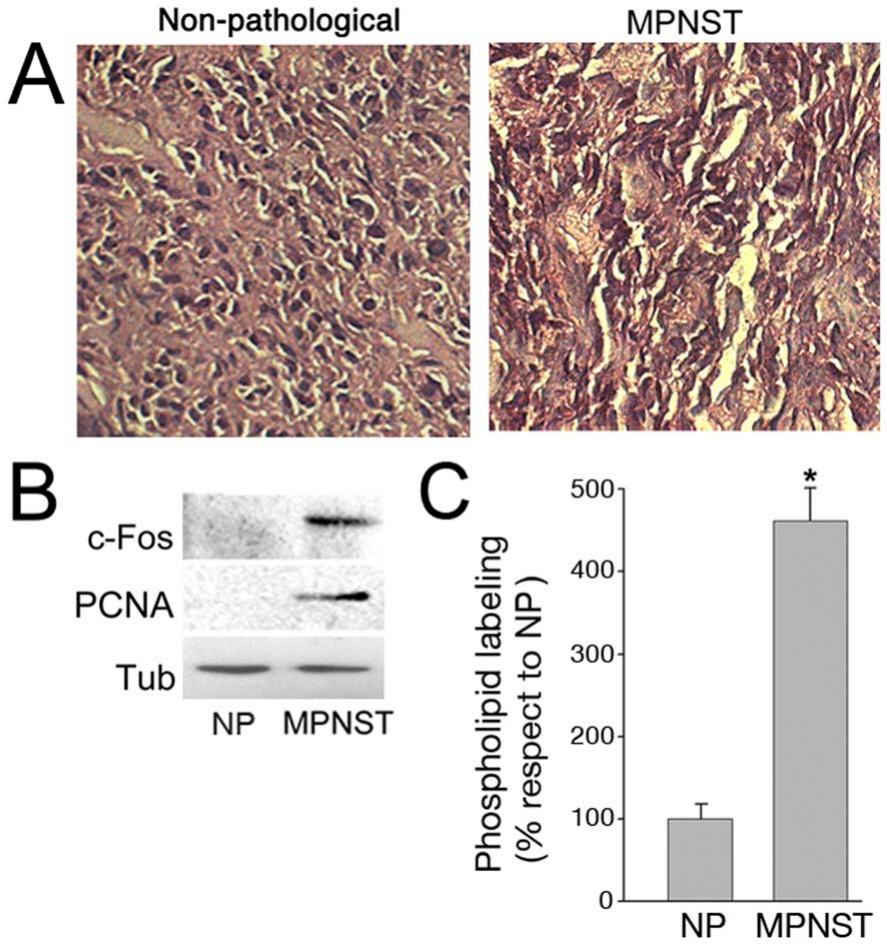
c-Fos expression is abundant and phospholipid synthesis is activated in a MPNST from a patient with NF1 syndrome. (**A**) Histological examination of a MPNST and a non-pathological specimen excised from an NF1patient, stained with haematoxylin/eosin. Note the neoplastic cells in the MPNST contrasting with the non-pathological (NP) tissue. (**B**) WB for c-Fos and PCNA of the samples shown in (A). Note the lack of c-Fos and PCNA expression in the normal tissue (NP). Tubulin was used as a loading control. (**C**) Phospholipid synthesis capacity in TH from the MPNST and the NP sample. Results are the mean % ± SD of 2 experiments performed in triplicate. TH values from NP were taken as 100%. Phospholipid synthesis activity in the MPNST was >4-fold that in NP; *p<0.005.

We next examined the presence of c-Fos in a MPNST excised from a human NF1 patient and in normal tissue excised adjacent to this tumor. The histological features of each sample are depicted on Figure 6A in which the presence of malignant-neoplastic cells are clearly observed in the transformed tissue respect to the non transformed one. Both c-Fos and PCNA expression are abundant in the MPNST contrasting with the non-detectable levels of these proteins observed in the normal nerve (Fig. 6B). Again, c-Fos expression appears tightly correlated with the proliferative state of the tissue: it is not detectable or absent in normal tissue and up-regulated in the tumor one. Finally, the capacity of both samples to synthesize phospholipids was measured *in vitro*. As expected, TH phospholipid synthesis was >4-fold higher in the MPNST respect to the normal sample (Fig. 6C).

## Discussion

Previous results disclosed a dual function of c-Fos that is topologically and temporarily separable: it first releases the genomic program for nuclear control of cell growth and then sustains it *via* its cytoplasmic association to the ER and activation of phospholipid synthesis [8], [10]. Since the above mentioned results were obtained from cells in culture, the role of c-Fos as a stimulator of membrane biogenesis in events associated to cell growth an proliferation must necessarily be confronted with results obtained using experimental paradigms that include the complex environment in which in vivo tumor development occurs.

Examination of an array of human brain tumors consistently showed abundant cytoplasmic, ER-associated c-Fos expression. Moreover, microsomal membranes from non fixed human malignant brain tumors showed activated phospholipid synthesis; this capacity was lost upon removal of c-Fos by washing with 1M KCl, but was recovered upon addition of recombinant c-Fos. Similar results were found in a MPNST in which abundant c-Fos expression together with a more than 4-fold increase in phospholipid synthesis activity was observed when compared to non-pathological tissue.

Tumor growth dependence on cytoplasmic c-Fos expression was further confirmed in tumor-bearing NPcis mice which consistently show elevated expression of ER-associated c-Fos in brain cortex and in proliferating cells of PNS and CNS tumors. c-Fos antisense oligonucleotide infused intra-cranially reduced c-Fos expression and cell proliferation. Moreover, no tumor development occurred in NPcis *fos*(−/−) mice in spite of the fairly conserved levels of AP-1 content in brain tissue from all animals irrespective of their c-Fos content. Thus, *in vivo* results in this model disclose the dependence of CNS and PNS tumor growth on cytoplasmic c-Fos expression and phospholipid synthesis activation. Also, compared to non-pathological tissue, human brain tumors evidenced abundant ER-associated c-Fos expression, and more than 2-fold activated phospholipid synthesis.

Diverse studies have suggested that, as a transcription factor, c-Fos is indispensable for proliferation. However, homozygous *fos*(−/−) mice are viable, morphologically normal but grow to a smaller final size than their counterpart WT or *fos*(+/−) animals [25]. This poses the question of why mitogenic stimulation of cells results so effective in inducing the expression of *c-fos* [1], [4] and yet c-Fos absence affects animal size rather than cell proliferation. Noteworthy is the observation that in these *fos*(−/−) mice another component of the AP-1 family, Fra-1, rescues c-Fos functions in the growth process when its expression is controlled by the c-Fos promoter but fails to substitute c-Fos in inducing expression of target genes in *fos*(−/−) fibroblasts [27]. Interestingly, the basic domain of c-Fos (BD that comprises amino acids 139–159), which is essential to achieve phospholipid synthesis activation [8], [9] is highly homologous to the BD of Fra-1, differing only in two conservative amino acid substitutions. This predicts the BD of Fra-1 as capable of activating phospholipid synthesis when Fra-1 is expressed and should, in consequence, support normal growth rates. Preliminary data from our laboratory have shown that this is indeed so (G Castro & BL Caputto, unpublished data). However, this is not the case in *fos*(−/−) animals probably because as in WT mice, only very small overlapping expression patterns of these proteins is observed when each gene is controlled by its own promoter [27]. So, even if AP-1 c-Fos may trigger the genomic events required for proliferation and growth, this activity must be exerted by other AP-1 complexes in the *fos(*−/−) mice.

At present there is abundant information about genomic events that underlie uncontrolled growth of tumor cells [28]. On the contrary, reports on the pleiotropic changes that necessarily accompany cell growth and proliferation are scarce. Although the elucidation of the molecular mechanism is still in progress, c-Fos is emerging as an inducible protein capable of activating the metabolic machinery of lipid synthesis for membrane biogenesis to accompany growth. Nevertheless, it is clear that not all enzymes of a lipid pathway must be activated to attain an overall lipid activated state [9], [29]. Regarding the activation mechanism, two different mechanisms seem at first sight feasible: one is that c-Fos interacts directly with the enzymes that it activates and the other is that c-Fos interacts with other ER components modifying the microenvironment of the enzymes that leads to their activation. In fact, both possibilities have experimental data that support them [9], [30]. In either case, the association of c-Fos to ER components activates for example the enzyme Glusosylceramide synthase by increasing its Vmax without substantially modifying its Km [9]. Having found that c-Fos deletion mutants that contain the NH_2_-terminus up to BD of the full length protein (aa 1–159) are capable of activating lipid synthesis to similar rates as c-Fos [8], [9] and that the phosphorylation of tyrosine residues 10 and 30 precludes c-Fos/ER association and consequently lipid synthesis activation [10] suggests that the NH_2_-terminus participates in associating c-Fos to ER components whereas BD would promote the activation of the corresponding enzymes.

The data available at present allow us to hypothesize a shared activation mechanism in response to high-rate membrane biogenesis demands of cells irrespective of these demands arising from physiological [5]–[9] or pathological situations as shown herein. Furthermore, not only tumor-associated pathologies show ER-associated c-Fos: in spinal cord of rats sensitized to develop experimental allergic encephalomyelitis in which positive reactive gliosis is promoted [31], c-Fos expression is also increased and ER-associated (Fig. S2). In support of this hypothesis it should be noted that herein it is shown that the addition of c-Fos both to a non-pathological brain preparation obtained from a GM-patient as well as to the tumor sample stripped of associated proteins (Fig. 2) results in an activated rate of phospholipid synthesis.

The cytoplasmic regulatory role of c-Fos is not the only AP-1 independent activity of an early response gene. c-Jun was also shown to protect cells from apoptosis independently of its AP-1 activity [32]. More recently, an additional cytoplasmic activity for *p53*, another well characterized transcription factor capable of triggering apoptosis has been described [33]. Consequently, the concept that the biological activities of these proteins results from the combination of their nuclear and cytoplasmic activities is worth considering [34]. Therapies directed towards blocking phospholipid synthesis activation by blocking c-Fos expression are promising moreover if it is considered that this protein is normally down regulated in healthy nervous tissue but becomes up-regulated during and is causally related to cancer progression.

## Materials and Methods

### Animals

*Nf1;Trp53 cis* (NPcis) mice were a kind gift from Tyler Jack's lab (Massachusetts Institute of Technology, USA). To maintain our animal colony, founders were bred to wild-type mice and progeny scored by PCR genotyping for both mutations on the same chromosome in *cis* using the primer sequences described [18].

*fos*(+/−) mice from Jackson Laboratories [25] were inbred to obtain *fos*(−/−) animals. NPcis animals were mated to *fos*(+/−) mice to obtain NPcis, *fos*(+/−) mice which were then mated with *fos*(+/−) mice to obtain NPcis, *fos*(−/−) mice. The wild-type allele of *c-fos* was amplified with the primers FKO1 (5′-GAGCAACTGAGAAGACTGGATAGAGCCGGC-3′) and FKO2 (5′-GGAGAGCCCATGCTGGAGAAGGAGTCG-3′); FKO2 and PN1 primers (5′-GGCGAGGATCTCGTCGTGACC-3′) were for *c-fos* mutant allele scoring. When comparing tumor burden, only male NPcis, *fos*(+/+), *fos*(+/−) or *fos*(−/−) were considered to avoid gender-promoted differences. All mice were on a C57BL/6J background and grown under standard conditions.

### Phospholipid Synthesis Determination

In vitro phospholipid synthesis capacity of tumors or sub-cellular fractions was performed as described previously [8]; when stated, recombinant His-tagged c-Fos (1 ng/µg of initial TH protein), obtained as in [30], was added suspended in 300 mM imidazole/8 M urea; control incubates contained an equal volume of vehicle. TH's prepared in H_2_O plus protease inhibitor cocktail (Sigma-Aldrich, St. Louis, MO, USA) were centrifuged for 1h at 100,000xg and separated into MF and SF. For stripping of TH, prior to centrifugation, TH's were made 1M with KCl, left to stand for 10 min, centrifuged at 100,000xg for 1h to separate into MF and SF. MF was re-suspended in the initial volume of H_2_O plus protease inhibitor cocktail.

Reactions were initiated by addition of 100 µg of TH, or the corresponding protein recovered in MF or stripped fractions re-suspended in incubation buffer, and stopped by addition of trichloroacetic acid – phosphotungstic acid (5%-0.5% final concentration). ^32^P-phospholipid labeling was quantified as described previously [35].

### Protein Quantification and Western Blot (WB) Analysis

10 µg of protein from tissue samples in H_2_O plus protease inhibitor cocktail were subjected to SDS-PAGE on 12% polyacrylamide gels as described [8]. Blocked membranes were incubated with rabbit anti c-Fos antibody (Santa Cruz Biotechnology, Santa Cruz, CA, USA dilution 1/5000), mouse DM1A mAb raised against α-Tubulin (Sigma-Aldrich, dilution 1/5000) or mouse anti PCNA antibody (Santa Cruz Biotechnology, dilution 1/1000), washed twice for 15 min in PBS-Tween and incubated with secondary biotin-conjugated antibody (Vector Laboratories, Burlingame, CA, USA, dilution 1/15000) raised against each corresponding primary antibody followed by incubation with streptavidin-peroxidase conjugated antibody (Amersham, Little Chalfont, Buckinghamshire, UK dilution 1/60000). Immunodetection was performed using ECL plus (Amersham) and protein concentration determined using Bradford Protein Assay (BioRad, Hercules, CA, USA).

### Electrophoretic Mobility Shift Assay (EMSA)

Nuclear extracts prepared as described by Wang et al. [36] were stored at -70°C until use. A double-stranded AP-1 oligonucleotide 5′-CGCTTGATGAGTCAGCCGGAA-3′ containing a TGAGTCA consensus sequence (Promega, Madison, WI, USA) was end-labeled with γ^ 32^P-ATP (Amersham) using T4 polynucleotide kinase. AP-1 binding reaction, AP-1 competition assay, and electrophoresis in non-denaturing polyacrylamide gels were performed as described by the manufacturer (Promega).

### Animal Treatment

NPcis mice with clear signs of peripheral nerve tumor burden were anesthetized with chloral hydrate (50 µgr/gr animal weight), a cannula (Alzet, Cupertino, CA, USA, Brain Infusion Kit II) was inserted into the right caudate putamen (CP) and connected to a osmotic minipump (Alzet, Model 2004) implanted under the neck skin to ensure continuous administration of the desired solution (350 nmol/7.8 µl DMEM/day, for 28 days). Minipumps were filled with *c-fos* mRNA Morpholino modified antisense (c-Fos ASO: 5′-GCGTTGAAACCCGAGAACATCATGG-3′) or sense oligonucleotide (c-Fos SO: 5′-GGTACTACAAGAGCCCAAAGTTGCG-3′) (Gene Tools, Pilomath, OR, USA).

All experiments were performed in agreement with the standards stated in the Guide to the Care and Use of Experimental Animals published by the Canadian Council on Animal Care and approved by the local animal care committee (Facultad de Ciencias Químicas, Universidad Nacional de Córdoba, Argentina, Exp. 15-99-39796).

### Immunohistochemistry

Anesthetized animals were perfused intracardially with 4% para-formaldehyde/PB 0.1 M. Excised brains and peripheral tumors were immersed in PB 0.1 M/30% sucrose and cryo-sectioned at 30 µm thickness. Sections were blocked in 4% BSA/0.3% Triton X100 in PBS 10 mM, stained with rabbit anti c-Fos (Santacruz Biotechnology, dilution 1/5000) or rabbit anti PCNA (Santacruz Biotechnology, dilution 1/5000) antibodies, diluted in blocking solution for 48 hours at 4°C and incubated with anti-rabbit biotinilated secondary antibody diluted in blocking solution (Vector, dilution 1/300) for 1h at room temperature. Detection was performed with the ABC kit (Vector) using DAB (Sigma-Aldrich) as peroxidase substrate. Slides were visualized on an Axiovert-200 (Carl Zeiss, Oberkochen, Germany) microscope and images obtained with a MicroMax CCD camera (Princeton Instruments, Trenton, NJ, USA).

### Human Tumor Samples

Fixed human brain tumor sections and matched benign specimens were from Ambion (Austin, TX, USA). Non-fixed human brain tumor and matched benign specimens were from patients that gave their physicians a written consent on forms approved by the corresponding Research Ethics Board of the Hospital San Roque and Clínica Vélez Sarsfield, Córdoba, and MPNST's at Hospital de Clínicas, Buenos Aires, Argentina. Samples were processed anonymous.

### Tissue Immunofluorescence

Specimens were de-waxed in xylene, re-hydrated, treated with target retrieval solution (DakoCytomation, Glostrup, Denmark) at 95°C for 30 min and blocked with 1% BSA/0.1% Tween 20 (v/v) in 10 mM PBS (blocking buffer). Primary antibodies diluted in blocking buffer as follows: rabbit anti c-Fos (Sigma-Aldrich, dilution 1/300), goat anti PCNA (Santacruz Biotechnology, dilution 1/300), mouse anti GFAP (Chemicon, Temacula, CA, USA, dilution 1/1000) and goat anti calnexin (Santacruz Biotechnology, dilution 1/300) antibodies were incubated overnight at 4°C. Anti goat Alexa 488, anti rabbit Alexa 546 and anti mouse Alexa 688 (Molecular Probes, Eugene, OR, USA) secondary antibodies were applied (dilution 1/500); slides were mounted in Prolong Antifade (Molecular Probes) and visualized on a confocal laser scanning LSM Pascal 5 microscope (Carl Zeiss). LSM 5 Image Browser software was used for image processing.

### Statistical Analysis

Statistical analysis was performed by Student's two tailed t test using Infostat software (Universidad Nacional de Córdoba, Argentina). Data are reported as mean ± SD. Differences were considered statistically significant at p<0.005.

## Supporting Information

Figure S1 c-Fos is abundantly expressed and co-localizes with the ER marker calnexin in embryonic mouse brain. Expression of c-Fos (red) and the ER marker calnexin (green) were determined in brain samples from 18 day old embryos and from adult animals. Note c-Fos/ER co-localization as evidenced in the merged image to the right.
(1.02 MB DOC)

Figure S2 c-Fos is abundantly expressed and co-localizes with the ER marker calnexin in spinal cord slices from rats with experimental allergic encephalomyelitis. Expression of c-Fos (red), the ER marker calnexin (green) and the reactive gliosis marker GFAP (grey) were determined in spinal cord slices from adult rats with clear symptoms of having developed experimental allergic encephalomyelitis [37] and littermate controls. The last column is the merge of the two first micrographs and clearly shows c-Fos/ER co-localization in the EAE samples that evidence reactive astrocytes as determined by GFAP immunostaining.
(0.39 MB DOC)
