# Supplementary material for: Growth of Peripheral and Central Nervous System Tumors Is Supported by Cytoplasmic c-Fos in Humans and Mice
Source: PLoS One. 2010 Mar 4;5(3):e9544. doi: 10.1371/journal.pone.0009544 (PMC2832012; doi:10.1371/journal.pone.0009544)
Supplement: Figure S2 — c-Fos is abundantly expressed and co-localizes with the ER marker calnexin in spinal cord slices from rats with experimental allergic encephalomyelitis. Expression of c-Fos (red), the ER marker calnexin (green) and the reactive gliosis marker GFAP (grey) were determined in spinal cord slices from adult rats with clear symptoms of having developed experimental allergic encephalomyelitis [37] and littermate controls. The last column is the merge of the two first micrographs and clearly shows c-Fos/ER co-localization in the EAE samples that evidence reactive astrocytes as determined by GFAP immunostaining. (0.39 MB DOC) [file pone.0009544.s002.doc]

**c-Fos Calnexin GFAP c-Fos/Calnexin**
