# Supplementary material for: Growth of Peripheral and Central Nervous System Tumors Is Supported by Cytoplasmic c-Fos in Humans and Mice
Source: PLoS One. 2010 Mar 4;5(3):e9544. doi: 10.1371/journal.pone.0009544 (PMC2832012; doi:10.1371/journal.pone.0009544)
Supplement: Supplementary file 2 [file pone.0009544.s001.doc]

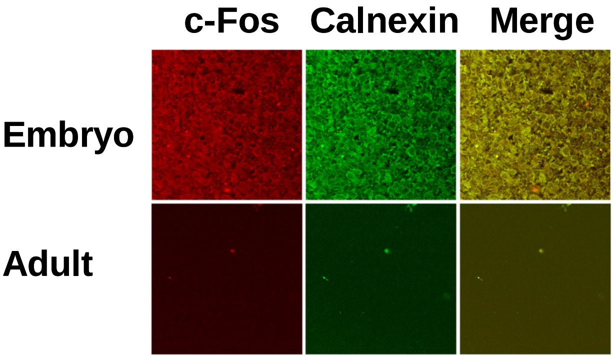
c-Fos
Calnexin
Merge
Embryo
Adult
